# Disease Severity, Fever, Age, and Sex Correlate With SARS-CoV-2 Neutralizing Antibody Responses

**DOI:** 10.3389/fimmu.2020.628971

**Published:** 2021-01-29

**Authors:** Stephan Schlickeiser, Tatjana Schwarz, Sophie Steiner, Kirsten Wittke, Nabeel Al Besher, Oliver Meyer, Ulrich Kalus, Axel Pruß, Florian Kurth, Thomas Zoller, Martin Witzenrath, Leif Erik Sander, Marcel A. Müller, Carmen Scheibenbogen, Hans-Dieter Volk, Christian Drosten, Victor M. Corman, Leif G. Hanitsch

**Affiliations:** ^1^ Institute of Medical Immunology, Charité - Universitätsmedizin Berlin, Corporate Member of Freie Universität Berlin, Humboldt-Universität zu Berlin, and Berlin Institute of Health, Berlin, Germany; ^2^ Berlin Institute of Health Center for Regenerative Therapies (BCRT), Charité - Universitätsmedizin Berlin, Berlin, Germany; ^3^ Institute of Virology, Charité-Universitätsmedizin Berlin, Humboldt-Universität zu Berlin, Berlin Institute of Health, and German Centre for Infection Research (DZIF), Partner Site Charité, Berlin, Germany; ^4^ Institute of Transfusion Medicine, Charité - Universitätsmedizin Berlin, Berlin, Germany; ^5^ Department of Infectious Diseases and Respiratory Medicine, Charité - Universitätsmedizin Berlin, Corporate Member of Freie Universität Berlin, Humboldt-Universität zu Berlin, and Berlin Institute of Health, Berlin, Germany; ^6^ Department of Tropical Medicine, Bernhard Nocht Institute for Tropical Medicine, Hamburg, Germany; ^7^ German Center for Lung Research (DZL), Partner Site Charité, Berlin, Germany; ^8^ Berlin Center for Advanced Therapies (BeCAT), Charité – Universitätsmedizin Berlin, Berlin, Germany

**Keywords:** COVID-19, SARS-CoV-2, immunodeficiency, antibody deficiency, coronavirus disease 2019, convalescent plasma, SARS-CoV-2 neutralizing antibody, plaque reduction neutralization test

## Abstract

Clinical trials on the use of COVID-19 convalescent plasma remain inconclusive. While data on safety is increasingly available, evidence for efficacy is still sparse. Subgroup analyses hint to a dose-response relationship between convalescent plasma neutralizing antibody levels and mortality. In particular, patients with primary and secondary antibody deficiency might benefit from this approach. However, testing of neutralizing antibodies is limited to specialized biosafety level 3 laboratories and is a time- and labor-intense procedure. In this single center study of 206 COVID-19 convalescent patients, clinical data, results of commercially available ELISA testing of SARS-CoV-2 spike-IgG and –IgA, and levels of neutralizing antibodies, determined by plaque reduction neutralization testing (PRNT), were analyzed. At a medium time point of 58 days after symptom onset, only 12.6% of potential plasma donors showed high levels of neutralizing antibodies (PRNT50 ≥ 1:320). Multivariable proportional odds logistic regression analysis revealed need for hospitalization due to COVID-19 (odds ratio 6.87; *p*-value 0.0004) and fever (odds ratio 3.00; *p*-value 0.0001) as leading factors affecting levels of SARS-CoV-2 neutralizing antibody titers in convalescent plasma donors. Using penalized estimation, a predictive proportional odds logistic regression model including the most important variables hospitalization, fever, age, sex, and anosmia or dysgeusia was developed. The predictive discrimination for PRNT50 ≥ 1:320 was reasonably good with AUC: 0.86 (with 95% CI: 0.79–0.92). Combining clinical and ELISA-based pre-screening, assessment of neutralizing antibodies could be spared in 75% of potential donors with a maximal loss of 10% of true positives (PRNT50 ≥ 1:320).

## Introduction

Convalescent plasma therapy has been advocated since the beginning of the global spread of SARS-CoV-2 (1). Historically, convalescent plasma was applied in different diseases, including influenza, SARS, and MERS (2–4). Rapid availability and low costs of convalescent plasma therapy have spurred researchers worldwide to focus on this treatment approach. Alone in the US, convalescent plasma was used in >40,000 COVID-19 patients generating strong safety data (5). While observational findings in cases or smaller cohorts appeared promising, reporting improved survival, radiological resolution and viral load (6–8), data from two randomized controlled trials in China and the EU, were terminated prematurely, underpowered and unable to prove clinical benefit (9, 10). In a recently completed randomized controlled trial from India the majority of patients (~70%) received plasma with low levels of SARS-CoV-2 neutralizing antibodies (<1:80) (11). However, subgroup analyses report a dose-response relationship between convalescent plasma neutralizing antibody level and mortality, suggesting that treatment with high levels of neutralizing antibodies is likely to be more beneficial and advocating the administration of accordingly selected plasmas in clinical trials (12). Based on pathophysiological considerations and first case reports, it is expected that in particular patients with deficient antibody production may be more likely to benefit from convalescent plasma treatment (13, 14).

Identifying adequate donors is of critical importance for any ongoing trials and centers actively recruiting convalescent plasma donors. Collection of convalescent plasma is highly regulated by national authorities requiring, e.g., different periods for quarantine before convalescent plasma donation and for storage before approval for use in COVID-19 patients. Levels of neutralizing antibodies required for prevention or treatment in COVID-19 remain to be determined. However, based on general pathophysiological considerations it seems plausible to use higher levels of antibodies and FDA and EMA advocate levels of at least 1:80 to 1:160 and preferably higher titers (15). Unfortunately, evaluating neutralizing antibody capacities requires highly specialized expertise, availability of a biosafety level 3 laboratory and is time- and labor-intense. Consequently, and despite significant variability between different commercially available ELISA tests, these tests are applied frequently as a surrogate marker for the levels of neutralizing antibodies (16).

In order to assess convalescent plasma therapy in COVID-19, characterization of convalescent plasma by neutralizing antibody capacities is indispensable for interpretation of safety and efficacy. Preference should be given to donors of COVID-19 convalescent plasma with the higher neutralizing antibody response. Multiple assays for the detection of anti-SARS-CoV-2 antibodies are available, including several systems for high-throughput testing (17–20). These assays, although not of major importance in standard care settings, but can support clinicians to assess immune response in patients and are an important tool in epidemiological studies (21). The gold-standard to assess the neutralizing capacity of serum antibodies is the plaque-reduction neutralization test by using wild type virus isolates. However, this test needs access to wild-type isolates and BSL3 capacity, including trained personnel. Recently, PRNT surrogate assays, such as pseudotype-based neutralization assays (22) and surrogate virus neutralization test (sVNT) were established showing promising performance (23, 24). However, algorithms without multiple and complex test systems may streamline the identification process of adequate plasmas donors.

In this single center study, we characterized potential COVID-19 convalescent plasma donors for presence of IgG- and IgA-antibodies to the S1 domain of the SARS-CoV-2 spike (S) protein by ELISA and for neutralizing antibody capacity determined by plaque reduction neutralization test (PRNT). Furthermore, by correlating these results with clinical data we aimed to identify a clinical vignette that would help to improve the donor selection process. We prefer donors with a PRNT50 of ≥1:320 enabling to achieve a neutralizing antibody titer (PRNT50) of >1:40 with the administration of 440 ml of convalescent plasma in recipients with <75 kg body weight or 660 ml of convalescent plasma in recipients with a body weight of 75-110 kg (calculation example assuming 40 ml plasma volume per kg body weight: 70 kg × 40 ml = 2800 ml; 440 ml of convalescent plasma/(2800 + 440 ml) = 0.14; PRNT50 of 1:320 × 0.14 = 1:43 in the recipient).

## Methods

### Human Subjects and Serum Samples

In April 2020 we started screening patients who recovered from mild to moderate COVID-19 for convalescent plasma donation. Potential plasma donors were selected in agreement with German national plasma donation guidelines. Required age range is 18–60 years in first time donors and 18–68 in experienced plasma donors, individual exceptions due to extraordinary fitness and in absence of relevant comorbidities can apply. According to national guidelines, convalescent subjects required also to be clinically asymptomatic after COVID-19 disease for at least 4 weeks prior to plasma donation. The use of blood from healthy human subjects and from COVID-19-convalescent subjects was approved by the Institutional Review Board at Charité - Universitätsmedizin Berlin (EA2/092/20 and EA2/066/20) (25). All patients enrolled gave written informed consent in person. Convalescent subjects had a history of SARS-CoV-2 infection confirmed by a positive RT-PCR from pharyngeal swab. Eligibility of potential donors was checked according to national regulatory guidelines for plasma donors (Richtlinie Hämotherapie) (26). Clinical data was collected using a questionnaire for clinical symptoms including fever, dyspnea, cough and anosmia/dysgeusia as well as for requirement for hospitalization due to COVID-19. For serologic and neutralization testing, 9-ml blood from each donor were collected in serum tubes. Serum tubes were centrifuged at 1,500 g and 20°C for 15 min and aliquoted into 500- to 1,000-μl aliquots and stored at −20°C until further processing.

### Enzyme-Linked Immunosorbent Assay for Anti-SARS-CoV-2 S1-IgG and -IgA

For the detection of IgG and IgA to the S1 domain of the SARS-COV-2 spike (S) protein, anti-SARS-CoV-2 assay was used according to the manufacturer´s instructions (Euroimmun, Lübeck, Germany). Serum samples were tested at a 1:101 dilution using the fully EUROIMMUN Analyzer. Optical density (OD) ratios were calculated by dividing the OD at 450 nm by the OD of the calibrator included in the kit. The calculated OD ratios can be used as a relative measure for the concentration of antibodies in the serum. For IgG and IgA response, an OD ratio < 1.1 was considered to be non-reactive.

### Plaque Reduction Neutralization Test for SARS-CoV-2

PRNTs for SARS-CoV-2 were performed as previously described (27, 28). Briefly, Vero E6 cells were seeded in a 24-well plate format. Sera were diluted and mixed with 100 plaque forming units of SARS-CoV-2 (strain: SARS-CoV-2/human/DEU/BavPat2-ChVir984, NCBI GenBank Acc. No. MT270112.1). Each 24-well was incubated with serum-virus solution for 1 h at 37°C. After 1 h, supernatants were discarded, cells were washed once with PBS, and 1.2% Avicel solution in DMEM was added to the wells. After 3 days at 37°C, all supernatants were discarded and cells were fixed using a 6% formaldehyde/PBS solution and stained with crystal violet. Serum dilutions with a plaque reduction of 50% (PRNT50) are referred to as titers.

### Statistical Analysis

All analyses were conducted in R (version 4.0.2.) using the *Hmisc* (version 4.4-0) and *rms* packages (version 6.0-0). For proportional odds (PO) logistic regression on categories of neutralizing SARS-CoV-2 antibody titers PRNT50 values for 1:320 (10 donors), 1:640 (7 donors), and 1:1,280 (9 donors) were pooled into a single category (≥1:320) to gain sufficient per-category sample sizes and 3 of 206 clinical records were excluded due to missing entries in several variables. The full model was prespecified to include variables sex, age in years, days after positive SARS-CoV-2 RT-PCR testing, fever, dyspnea, cough, anosmia/dysgeusia, and requirement for hospitalization. For developing a predictive model, shrunken coefficients of the full model were first re-estimated by L2-penalized regression and the linear predictors approximated by ordinary linear regression and step-down backward selection of the top 4 important predictors with only minute loss of precision (R^2^ > 0.99 compared to full model). Correct estimates of the variance of the coefficients of the reduced model were calculated using equation 5.2 in (29).

A second PO logistic regression model was used to calculate L2-penalized regression coefficients of anti-SARS-CoV-2 S1-IgG OD ratios for higher SARS-CoV-2 neutralizing antibody titers. Coefficients and covariance matrices of both models were used to create spreadsheets (**Supplementary Data Sheet 2** and **3**) in large adaptation of an example given by (30). Receiver-operator characteristics were analyzed using the *pROC* package (version 1.16.2). Specificities at 0.95 target sensitivity and 95% confidence intervals (CI) for all performance measures were computed with 2,000 stratified bootstrap replicates. The true positive number after two consecutively performed tests A and B can be estimated by TP_AB_ = T × (Se_A_ × Se_B_ − covSe_AB_), with T the number of real cases and the final serial-test sensitivity which is given by the product of the individual tests’ sensitivities Se_A_ and Se_B_, adjusted by their conditional covariance covSe_AB_. The false positive number calculates as FP_AB_ = N × ((1 − Sp_A_) × (1 − Sp_B_) + covSp_AB_), with N the number of real negative cases, and Sp_A_, Sp_B_ the individual tests’ specificities and their covariance covSp_AB_. The final specificity of two consecutive tests can be estimated by Sp_AB_ = Sp_A_ + Sp_B_ − Sp_A_ × Sp_B_ − covSp_AB_ (31).

## Results

### Clinical Characteristics

Potential plasma donors contacted our clinic after being diagnosed with COVID-19. General suitability for plasma donation was evaluated before presentation at our clinic by telephone interview. In total, 206 potential convalescent plasma donors were assessed. In agreement with national recommendations, all convalescent patients had a symptomatic confirmed SARS-CoV-2 infection (mandatory positive SARS-CoV-2 test by RT-PCR testing in pharyngeal swab). In addition, patients required to be clinically asymptomatic after COVID-19 disease for at least 4 weeks.

All 206 donors were tested by ELISA for IgG and IgA against SARS-CoV-2 S1 spike and by plaque reduction neutralization test (PRNT50). In total, 54% of donors were female, and median age of donors was 37.4 years. Median time point of antibody testing was 53 days after positive SARS-CoV-2 PCR testing and 58 days after symptom onset. Patients were interviewed for clinical symptoms including fever, dyspnea, cough, and anosmia/dysgeusia. In addition, patients were asked to report requirement for hospitalization due to COVID-19.

Fever was reported by 109 donors (53%), dyspnea by 70 donors (34%), cough by 130 (63%), and anosmia/dysgeusia by 111 donors (54%). Hospitalization due to COVID-19 was reported by 19 donors (9%) (**Table 1**).

**Table 1:** Characteristics of COVID-19 convalescent plasma donors.

Total number of convalescent plasma donors^a^	206
Median age in years (range^b^)	37.4 (18–74)
Female sex (%)	111 (54%)
Median time point after positive SARS-CoV-2 RT-PCR testing in days (range)	53 (25–125)
Median time point after symptom onset in days (range)	58 (32–30)
**SARS-CoV-2 antibodies:**	
Median SARS-CoV-2 S1-IgG ratio	2.9
Median SARS-CoV-2 S1-IgA ratio	1.77
Median PRNT50 (titer)	1:40
Convalescent plasma donors with PRNT50 <1:20 (%)	33 (16%)
Convalescent plasma donors with PRNT50 ≥1:20–<1:40 (%)	39 (19%)
Convalescent plasma donors with PRNT50 ≥1:40–<1:80 (%)	38 (18%)
Convalescent plasma donors with PRNT50 ≥1:80–<1:160 (%)	40 (19%)
Convalescent plasma donors with PRNT50 ≥1:160–<1:320 (%)	30 (15%)
Convalescent plasma donors with PRNT50 ≥1:320 (%)	26 (13%)
	
**Clinical symptoms:**	
Convalescent plasma donors with fever (%)	109 (53%)
Convalescent plasma donors with dyspnea^c^ (%)	70 (34%)
Convalescent plasma donors with cough^c^ (%)	130 (63%)
Convalescent plasma donors with anosmia/dysgeusia^c^ (%)	111 (54%)
Convalescent plasma donors hospitalized for COVID-19 (%)	19 (9%)

a All donors were of Caucasian race/ethnicity, except a single Asian donor.

b Two male donors were 70 and 74 years, all else were less than 65 years old.

c Three donors had missing entries for dyspnea, cough and anosmia/dysgeusia.

### Detection of Anti-SARS-CoV-2 S1-IgG and IgA by ELISA

Despite positive SARS-CoV-2 RT-PCR result, IgG-and IgA-antibodies against S1 spike protein were only detectable in 89% of donors. Using the manufactures cut-off for SARS-CoV-2 spike ELISA with a ratio < 1.1, a total of 23 donors showed seronegativity for both (IgG(−)/IgA(−)), 42 donors were IgG(+)/IgA(−), six donors were IgG(−)/IgA(+).

### Detection of Neutralizing Antibodies by Plaque Reduction Neutralization Test (PRNT50)

No detectable neutralizing SARS-CoV-2 antibodies (PRNT50 < 1:20) were found in 33 donors (16%). A PRNT50 of ≥1:20–<1:40 was detectable in 39 donors (19%), a value of ≥1:40–<1:80 was found in 38 donors (18%), PRNT50 levels of ≥1:80–<1:160 were seen in 40 donors (19%), levels of ≥1:160–<1:320 in 30 donors (15%) and PRNT50 levels ≥ 1:320 were detected in 26 (13%) (**Table 1**).

### Correlation of Anti-SARS-CoV-2 S1-IgG and IgA With Levels of Neutralizing Antibodies (PRNT50)

**Figure 1:**
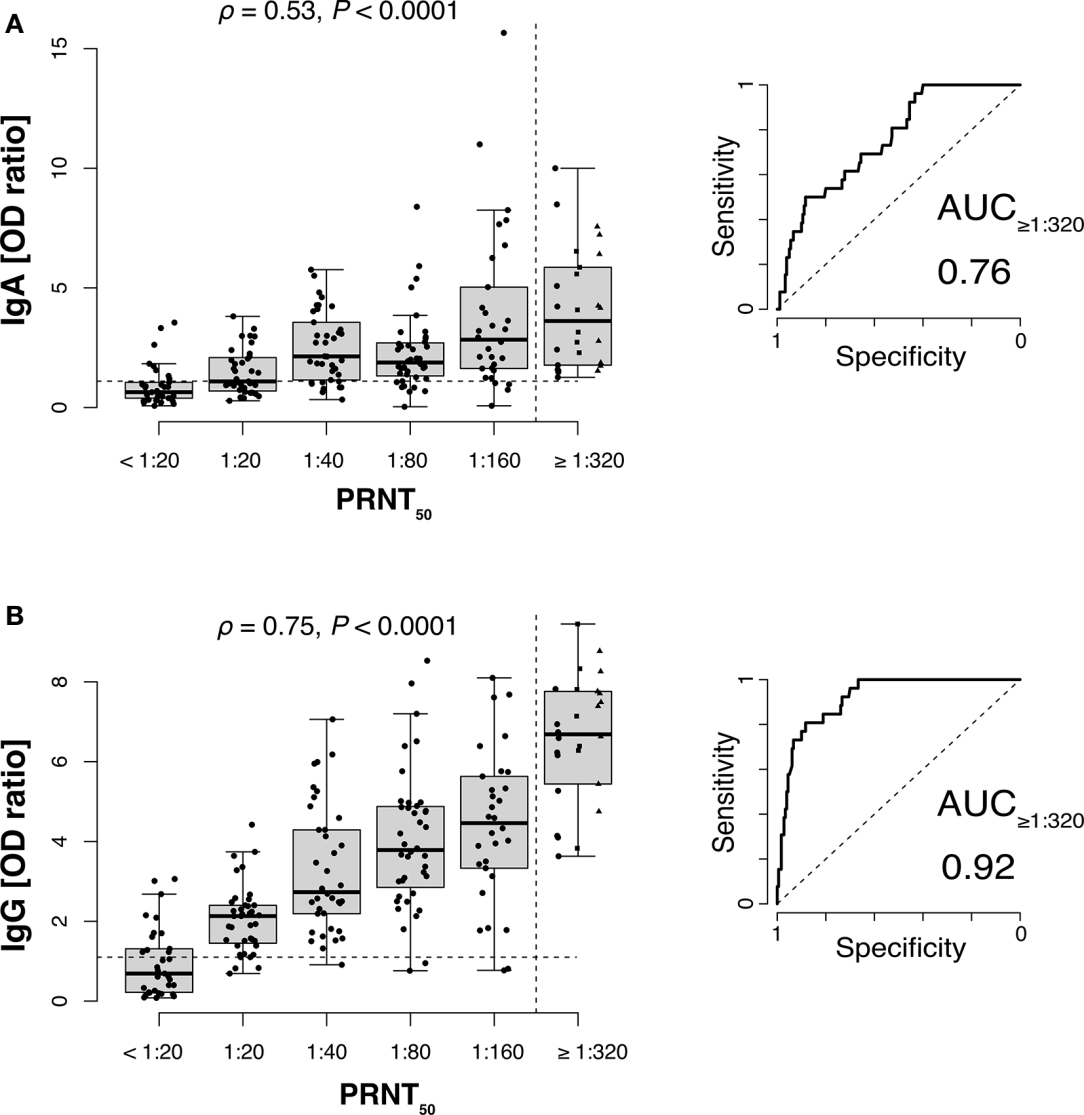
SARS-CoV-2 neutralizing antibody titers correlate with anti-S1 IgG and to a lesser extend with IgA antibodies. Plaque reduction neutralization test (PRNT_50_) titers of 206 plasma donors were correlated with anti-S1 IgA **(A)** and anti-S1 IgG **(B)** ELISA measurements (*ρ*, Spearman’s rank correlation coefficients). Values for 1:320, 1:640 (squares), and 1:1,280 (triangles) titers were pooled into a single category (≥1:320) to gain sufficient per-category sample sizes and to represent the recommended minimum titer for convalescent plasma therapy (dashed vertical line). Dashed horizontal lines indicate ELISA positivity cutoffs provided by the manufacturer EUROIMMUN). Right panels show receiver-operator characteristics (ROC curves) for prediction of a PRNT_50_ titer of at least 1:320 (AUC, area under the curve).

SARS-CoV-2 neutralizing antibody titers significantly correlated both with anti-S1 IgG and anti-S1 IgA antibodies (**Figure 1**). Receiver-operator characteristics (ROC curves) for prediction of a PRNT50 titer of at least 1:320 revealed only moderate discrimination when using anti-S1 IgA as predictor (AUC: 0.76 with 95% CI: 0.67–0.84, **Figure 1A**) whereas anti-S1 IgG performed very well (AUC: 0.92 with 95% CI: 0.87–0.96, **Figure 1B**).

**Figure 2:**
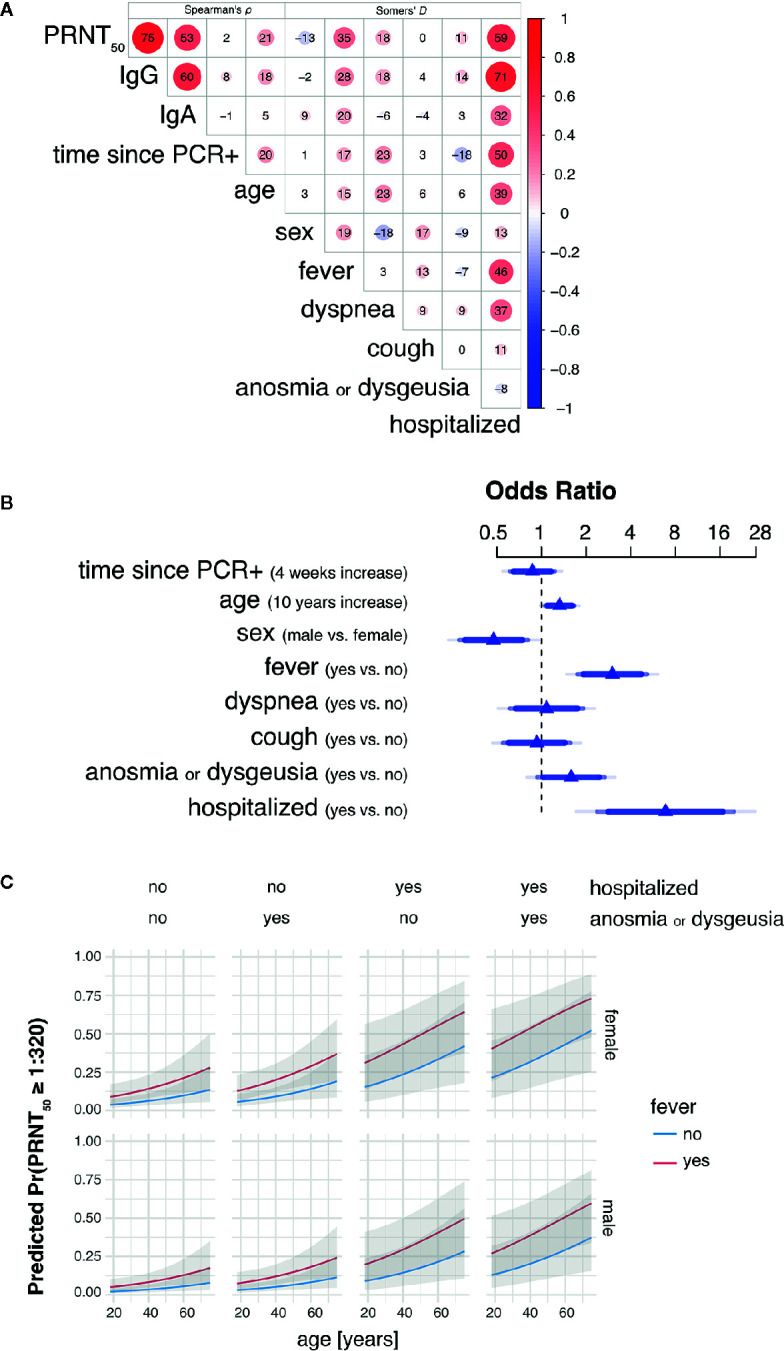
Hospitalization, fever and age are main predictors of high SARS-CoV-2 neutralizing antibody titers in convalescent plasma. **(A)** Heat plot representation summarizing bivariate rank correlation coefficients between all variables analyzed. Shown is only the upper triangle of the correlation matrix where correlation coefficients are given as Spearman’s *ρ* for continuous variables (four leftmost columns) and Somers’ *D* for bivariate correlations including at least one binary variable. Positive and negative correlations are colored in red and blue, respectively. Numbers within circles represent correlation coefficients expressed as percent while circle sizes are proportional to the absolute correlation coefficient values. **(B)** Adjusted odds ratios (OR) estimated by multivariable proportional odds (PO) logistic regression analysis are shown for each predictor of PRNT_50_ titer as response variable. OR of continuous variables are calculated for 4 weeks increase in time since PCR+ and 10 years increase in age. Blue of different transparencies indicates 90, 95, and 99% confidence levels. **(C)** Predicted probabilities for PRNT_50_ titers of at least 1:320 for varying predictor settings with 95% confidence bands. Shown are predicted probabilities for age ranging from 18 to 75 years in females (top row) and males (bottom row) by all possible combinations of hospitalization status with presence of anosmia or dysgeusia (columns) and fever (blue and red lines). Effects were estimated by a L2-penalized PO logistic regression model including all shown, most important variables age, sex, fever, anosmia or dysgeusia, and hospitalization.

Bivariate analyses using non-parametric rank correlation coefficients further revealed strong association of PRNT50 titers with presence of fever and hospitalization status, the latter of which positively correlated with other variables indicative of disease severity, such as fever, dyspnea, age, and time since positive PCR testing. A summarizing correlation matrix between all variables analyzed is shown by heat plot representation in **Figure 2A**.

### Multivariable Analysis of Clinical Features With Levels of Neutralizing Antibodies (PRNT50)

Multivariable PO logistic regression analysis revealed need for hospitalization for COVID-19 (odds ratio 6.87; *p*-value 0.0004) and fever (odds ratio 3.00; *p*-value 0.0001) as leading factors affecting levels of SARS-CoV-2 neutralizing antibody titers in convalescent plasma donors (**Table 2** and **Figure 2B**). Also increasing age (odds ratio 1.32; *p*-value 0.0165) and male sex (odds ratio 0.47; *p*-value 0.006) influenced neutralizing antibody titers. There was a trend toward significance for anosmia/dysgeusia (odds ratio 1.58; *p*-value 0.0847), while coefficients for time point since RT-PCR positivity, dyspnea and coughing were not significant in the model (**Table 2** and **Figure 2B**).

**Table 2:** Multivariable proportional odds logistic regression analysis of factors affecting SARS-CoV-2 neutralizing antibody titers in convalescent plasma.

Factor	χ^2^	d.f.	*P-value*	OR (95% CI)
Time since PCR+	0.65	1	0.4198	0.87 (0.61, 1.23)
Sex (male)*	7.55	1	0.0060	0.47 (0.28, 0.81)
Age*	5.75	1	0.0165	1.32 (1.05, 1.67)
Fever*	16.02	1	0.0001	3.00 (1.75, 5.14)
Dyspnea	0.07	1	0.7921	1.08 (0.61, 1.91)
Cough	0.08	1	0.7796	0.93 (0.55, 1.56)
Anosmia or Dysgeusia*	2.97	1	0.0847	1.58 (0.94, 2.67)
Hospitalized*	12.66	1	0.0004	6.87 (2.38, 19.88)
TOTAL	48.04	8	<0.0001	

Wald statistics and adjusted odds ratios are shown for each predictor of PRNT50 titer as response variable (d.f., degrees of freedom; OR, odds ratio; CI, confidence interval). Odds ratios of continuous variables are calculated for 4 weeks increase in time since PCR+ and 10 years increase in age. Asterisks indicate backward-selected variables for a final predictive model (**Figure 2C**).

### Model for Neutralizing Anti-SARS-CoV-2 Antibody Levels to Tailor an Individual Screening Strategy

Using penalized estimation, a predictive PO logistic regression model including the most important variables age, sex, fever, hospitalization, and anosmia or dysgeusia was developed (see **Supplementary Data Sheet 3**). The predictive discrimination for PRNT50 ≥ 1:320 was reasonably good with AUC: 0.86 (CI: 0.79–0.92). Probabilities for PRNT50 ≥ 1:320 were modelled to demonstrate effects of the individual predictors (**Figure 2C**). Both male and female donors showed an increase of predicted probability with increasing age. Female donors and fever during COVID-19 resulted in a higher predicted probability for PRNT50 ≥ 1:320. Predicted probability increased further when donors were hospitalized for COVID-19 and additionally developed anosmia or dysgeusia. The highest probabilities for PRNT50 ≥1:320 can therefore be expected in male and female patients aged 60 years or older with fever, previous hospitalization for COVID-19 and accompanying anosmia or dysgeusia.

In order to reduce labor and cost in performing plaque reduction neutralization testing, a clinical prediction model and anti-S1 IgG testing may be utilized with an appropriately defined sensitivity for preselection with minute loss of donors having potentially high neutralizing anti-SARS-CoV-2 antibody titers (see **Supplementary Material** section).

Using the clinical vignette with a classification cutoff at 0.95 target sensitivity, approximately, one in four positively screened donors is expected to have a titer ≥ 1:320, corresponding to a positive predictive value (PPV) of 0.27 (CI: 0.19–0.34) and a specificity of 0.62 (CI: 0.41–0.73). At 0.95 sensitivity, the anti-S1 IgG ELISA has 0.71 specificity (CI: 0.62–0.88) and a PPV of 0.32 (CI: 0.26–0.54), i.e., one in three donors tested above the corresponding cutoff IgG OD ratio of 3.83 will have a PRNT50 titer of 1:320 or higher.

Aiming on gain in pre-screening specificity, i.e., to further reduce numbers of false-positives that may enter PRNT, a serial testing strategy can be devised where anti-S1 IgG ELISA is performed only in donors that were positively classified in the clinical vignette. Accepting 10% loss of donors with PRNT50 ≥ 1:320 (i.e., 0.95 × 0.95 target sensitivity), conditional pre-selection would result in a sensitivity of 0.89 (CI: 0.79–0.98), a specificity of 0.85 (CI: 0.79–0.95) and a PPV of 0.46 (CI: 0.38–0.72) corresponding to 23 true positive (CI: 21–25) and 27 false positive (CI: 9–38) out of the 203 available donors in our study. Thus, if anti-S1 IgG ELISA testing was performed in donors pre-identified *via* the clinical vignette, analyzing PRNT could have been spared in 75% of the donors of this cohort with the given prevalence of 12.6% for PRNT50 titers ≥ 1:320.

## Discussion

With the ongoing SARS-CoV-2 pandemic, convalescent plasma continues to be a plausible treatment option, in particular for patients with impaired specific SARS-CoV-2 antibody response (e.g., primary and secondary immunodeficiency). Although data showing clear efficacy is still missing, there is an increasing body of evidence, that clinical benefit is linked to the neutralizing capacity of convalescent plasma (12, 32–34). Selecting plasma donors with high probability for higher levels of neutralizing antibodies is therefore of importance for any ongoing or future convalescent plasma trial.

While levels of neutralizing antibodies to MERS-CoV were reported to remain stable for > 2 years (35), for SARS-CoV-2, it is now recognized, that antibody levels are already declining after 3 months (36, 37). Therefore, identifying potential COVID-19 plasma donors is underlying additional time constraints.

In line with the highly variable disease course in COVID-19, also humoral immune response expresses a great heterogeneity. In this study higher levels of neutralizing antibodies (≥1:320 in PRNT50) were only detectable in 12.6% of donors. Despite positive RT-PCR testing for SARS-CoV-2 in nasopharyngeal swab, 11% of donors did not develop any detectable SARS-CoV-2 S1-IgG or -IgA antibodies and 16% no detectable neutralizing antibodies, respectively.

The availability of prescreening algorithms in order to increase the probability of selecting plasma donors with higher neutralizing antibody titers could support convalescent plasma collection.

The assessment of clinical convalescent donor characteristics is readily available and could serve as a “pre-test” filter. Our study showed that in COVID-19 convalescent plasma donors, clinical parameters of disease severity (i.e., need for hospitalization, fever and anosmia or dysgeusia) as well as sex and age correlate with levels of neutralizing antibodies against SARS-CoV-2. Using additional ELISA tests, donors with a given level of neutralizing antibodies, i.e., ≥1:320, may reliably be identified with appropriate cutoffs.

Depending on the available local resources for testing neutralizing antibody titers and depending on the availability of convalescent plasma donors, i.e., high or low prevalence regions, different sensitivities and specificities may be preferred. Prediction models may help to develop an individual screening strategy for potential convalescent plasma donors. In our cohort, combining clinical vignette and IgG-ELISA, both with a cutoff at target sensitivity of 95%, PRNT assays could be spared in 75% of initial donors with the accepted loss of 10% of true positives. Our observation of 13% high neutralizing antibodies (PRNT50 of ≥1:320) is in line with recent reports from smaller cohorts in the US and Europe, reporting 13%–14.5% with PRNT50 of ≥ 1:500 (38, 39). In contrast, a recent publication from Brazil showed a PRNT50 > 1:320 in approximately 38% of donors, however donors were tested earlier. Wendel et al, also highlighted that 36.2% of convalescent plasma donors remained SARS-CoV-2 RT-PCR positive within 28-48 days of recovery (40). Klein et al. detected neutralizing antibodies (>1:20) in 80% but did not report specifically on different titer dilutions (41).

Our results confirm that clinical severity (i.e., need for hospitalization) and age predicts levels of neutralizing antibody responses (41). In addition, we found that fever and anosmia/dysgeusia correlates with neutralizing antibody titers which is also supported by other studies (42–44). It remains inconclusive, if and how sex correlates with neutralizing antibody responses in COVID-19 patients. In general, females develop more profound adaptive immune responses against viral infections and vaccines than males which might translate into the observed sex-differences in SARS-CoV-2 pathogenesis and that may underly reduced disease vulnerability in women (45–48). In patients with moderate COVID-19, Takahashi et al. report (49) significantly higher T cell activation and a trend for higher SARS-CoV-2-specific antibody titers in female patients. While we and others found that female donors had a higher probability of high neutralizing antibodies (18, 50) or SARS-CoV-2 IgG antibodies (49, 51, 52), other reports had the opposite finding (38, 41, 53–56) or did not find a significance at all (39, 57, 58). These seemingly contradictory findings might be due to sampling or selection bias in individual studies but also to apparently different dynamics in the SARS-CoV-2 neutralizing antibody response in male and female donors (50, 59). Despite our finding of higher antibody responses in female donors, it is important to stress that female donors after pregnancy carry an elevated risk of HLA- and HNA-antibodies. These antibodies are associated with increased transfusion reactions resulting in the exclusion for plasma donation. Generally excluding female donors would therefore spare resource for these additional tests.

Our study has limitations. It is of cross-sectional nature, and the narrow time window of sample collection with regard to COVID-19 disease onset does not allow to analyze possible kinetics of antibody responses. Results of our study are limited to a specific ELISA test system recognizing antibodies against spike protein, so we cannot apply our observations as a general rule to other ELISA systems. Our analysis is not representative of the humoral immune response to SARS-CoV-2 in all patients but focuses on a rather homogeneous cohort of convalescent patients that would qualify as plasma donors. Although application of our prediction tool on a publicly available dataset (18) demonstrated acceptable accuracy (**Supplementary Data Sheet 4**), predictive performance may be worse in another clinical setting, e.g., due to a different case-mix, thus requiring external validation and potential recalibration of the prescreening model. In addition, no standardization of the different neutralizing antibody assays has been conducted. However, type of cell line, real virus or pseudovirus, count of viral particles, culture volumes and other factors are all likely to affect test results. It is therefore difficult to compare levels of (neutralizing) antibodies to SARS-CoV-2 from different studies.

The wide spectrum of antibody responses in convalescent plasma donors with mild COVID-19 disease remains an important issue. Our data support a clinical evaluation of COVID-19 symptoms as a pre-filter in order to identify convalescent plasma donors with potentially higher neutralizing antibody levels. The availability of prescreening algorithms in order to increase the probability of selecting plasma donors with higher neutralizing antibody titers could support convalescent plasma collection by sparing resources for labor-intense testing of neutralizing antibodies.

## Data Availability Statement

The original contributions presented in the study are included in the article/**Supplementary Material**. Further inquiries can be directed to the corresponding author.

## Ethics Statement

The studies involving human participants were reviewed and approved by Institutional Review Board at Charité Universitaetsmedizin Berlin (EA2/092/20 and EA2/066/20). The patients/participants provided their written informed consent to participate in this study.

## Author Contributions

SSc and LH made substantial contributions to conception and design. LH, KW, FK, and TZ made patient samples available. SSc, TS, VC, MM, and LH performed acquisition and analysis of data. SSc, TS, VC, and LH performed interpretation of data. TS, MM, CD, and VC performed analysis of neutralizing IgG and ELISA data. SSc, TS, VC, and LH wrote the article. SSt, KW, NAB, OM, FK, TZ, MW, LS, MM, CS, H-DV, and CD reviewed the manuscript critically for important intellectual content. All authors contributed to the article and approved the submitted version.

## Funding

Parts of this work were funded by the German Ministry of Health (Konsiliarlabor für Coronaviren) to CD and VC. The other researchers did not receive any specific grant from funding agencies in the public, commercial, or not-for-profit sectors. We acknowledge support from the Open Access Publication Fund of Charité – Universitätsmedizin Berlin.

## Conflict of Interest

MM and VC are named together with Euroimmun GmbH on a patent application filed recently regarding the diagnostic of SARS-CoV-2 by antibody testing.

The remaining authors declare that the research was conducted in the absence of any commercial or financial relationships that could be construed as a potential conflict of interest.

## Supplementary Material

Supplementary Data Sheet 1 ROC analysis for PRNT50 ≥1:320. This Excel file contains two spreadsheets with receiver-operator characteristics for using anti-SARS-CoV-2 S1-IgG OD ratio as predictor of PRNT50 titers ≥1:320, and for using the PO logistic regression model with a four-variable clinical vignette (sex, age, fever, anosmia/dysgeusia, and requirement for hospitalization) to predict PRNT50 titers ≥1:320, respectively. The spreadsheets list several performance measures for a range of possible thresholds as given by each donor’s predicted value. These may be used to identify classification cutoffs at a certain target value of a performance measure, for example a sensitivity of 95%.

Supplementary Data Sheet 2 Predictive PO model for ELISA IgG OD ratio. This Excel file contains spreadsheets to calculate probabilities of high PRNT50 titers using IgG OD ratio values as input. The first sheet provides a detailed description of how this may be used. In short, IgG OD ratio values as determined using Euroimmun anti-SARS-CoV-2 S1-IgG ELISA can be entered to calculate probabilities of higher PRNT50 titers in sheets for either of the categories “PRNT50>=320”, “PRNT50>=160”, “PRNT50>=80”, “PRNT50>=40”, or “PRNT50>=20”, respectively. For example, in “PRNT50>=320” an estimated probability higher than 0.06 (corresponding to IgG OD 3.83 for 95% target sensitivity) would qualify a sample for inclusion into final plaque reduction neutralization testing. For demonstration purposes, sheets are prefilled with data from this study.

Supplementary Data Sheet 3 Predictive PO model for clinical data. This Excel file contains spreadsheets to calculate probabilities of high PRNT50 titers using a clinical vignette with four important variables as input. The first sheet provides a detailed description of how this may be used. In short, patient characteristics (sex, age, fever, anosmia/dysgeusia, and requirement for hospitalization) can be entered to calculate probabilities of higher PRNT50 titers in sheets for either of the categories “PRNT50>=320”, “PRNT50>=160”, “PRNT50>=80”, “PRNT50>=40”, or “PRNT50>=20”, respectively. For example, in “PRNT50>=320” an estimated probability higher than 0.1046 (for 95% target sensitivity) would qualify a sample for inclusion into further anti-SARS-CoV-2 S1-IgG ELISA screening or final plaque reduction neutralization testing. For demonstration purposes, sheets are prefilled with data from this study.

Supplementary Data Sheet 4 Validation of Predictive PO using data from Padoan et al. Padoan et al. (18) have assessed the performance of different immunoassays for the detection of SARS-CoV-2 antibodies in comparison with neutralization activity. For 68 plasma donors an anti-spike IgG assay as well as PRNT testing was performed and results are publicly available in a dataset (60) which is used here to demonstrate the utility of the Excel prediction-tool. Data acquired at least 25 days after disease onset of 6 non-hospitalized and 22 hospitalized (12 moderate, 10 severe) recovered patients is used to calculate performance measures for the prediction of having a PRNT50 titer of ≥1:320. Accuracy (F-measure) was 0.79 for using the clinical vignette alone and 0.83 in combination with anti-Spike IgG.
